# Smallpox, with Its Prevention by General Vaccination

**Published:** 1894-03

**Authors:** 


### Editorial.

### SMALLPOX, WITH ITS PREVENTION BY GENERAL VACCINATION.

The extraordinary attention given by the daily papers of Atlanta recently to the subject of smallpox warrants some passing notice. It is a difficult matter for the public prints to deal with this disease dispassionately, owing to the great interests involved in providing for the welfare of the population and in averting the unfavorable impressions upon the trade of our merchants. But it is the province of medical men to look into the origin and progress of this contagious disease from a scientific standpoint.

The presence of a single case of variola calls for diligence in preventing its extension, and it is a most remarkable circumstance in connection with the existence of this disease in our city, that it should have been brought to the attention of our authorities by the report of a case which had been contracted here and developed at a distant point. Calculating the stage of incubation at from twelve to fifteen days and the progress of the eruption to a point for unmistakable diagnosis at a week more, we have the fact established of the existence of smallpox in Atlanta for a period of at least twenty days before it became known to our Board of Health. If the patient or patients affected during this time came under the care of a physician, it seems a strange oversight that the disease was not recognized and reported. The only plausible explanation is that it occurred in vaccinated persons and was mistaken for varicella or chickenpox, and yet was sufficient to propagate genuine variola to those unvaccinated.

It devolved upon me, at the request of the brother of the patient who left here with the seeds of the disease in his system, to visit the house from which he had gone and examine the cases on the evening of February 27th. I found a little girl about four years old, with a fully developed eruption of confluent smallpox and several cases with varioloid, while others were convalescing from this latter form of the disease.

I reported these facts by telephone to the President of the Board of Health and to the brother of the man who had taken the disease at this house.

As the building was completely isolated and contact with outsiders could be well secured by placing a guard around the house, it was determined to let the patients with other inmates remain secluded.

A physician was put in charge and vaccination of all who were not already attacked was carried out by him.

I saw these patients again on March 6th, and verified that the disease had run the usual course with desquamation, excepting a few scabs still remaining upon the hands and feet.

The cases of varioloid which had come under my observation at the previous visit were then convalescent, and no new cases had occurred. An infant at the breast that was vaccinated and re-vaccinated had not developed the disease, nor had the effects of the vaccine appeared. It is an interesting fact that the young man who was boarding at the house, when his brother contracted the disease, has been confined there ever since, and does not manifest any signs of disease or any effects of vaccination, though not vaccinated at any previous time.

A very close watch has been kept over this house and it is not expected that there will be any extension of the disease in that vicinity.

A number of cases of eruptive disease have been reported in the suburbs of the city, but none in the more populated central parts, and most of these cases have been considered as being chicken-pox, by the physicians in charge. Yet there is a notable feature in the fact of vaccination having been previously employed in all these cases of supposed varicella, and thus favoring the diagnosis of varioloid.

A case in point was presented before the Atlanta Society of Medicine at a recent meeting. The boy was white, about fourteen years, and had a temperature of 102.8° F., pulse 120 to the minute, and *papular* eruption scattered over face, neck, body, arms and legs, with several *papules* on the soft palate; old vaccine mark on arm. One of the city physicians pronounced this case chickenpox, and some of the members of the society concurred in this diagnosis. But the majority, with whom I agreed, regarded it as varioloid, and that it was likely to propagate smallpox if not isolated. A report to this effect was made to the President of the Board of Health.

Since the above report was made I was requested by a gentleman interested in knowing the nature of a suspected case at 321 Wheat street, to visit the patient and let him know the result of my examination. Upon going to see the case with another physician, he informed me that the same city physician who had pronounced the case before the medical society to be chickenpox declares this also to be a case of varicella. The eruption of a pustular form was well defined over the face, chest and arms, with evident eruption in the fauces and throat, causing difficulty in deglutition, and it was not deemed necessary to carry the examination further to make out a clear diagnosis of variola. There was even running together of the pustules in many places, bringing the case under the classification of confluent smallpox. One of the Board of Health went out afterwards to examine this patient and concurred in my view of the case, yet it was stated in the report of the Board of Health as a suspected case. A correct view is given in the following Board of Health bulletin:

ATLANTA, GA., March 13, 1894.—The case removed from 321 Wheat street is one of unmistakable confluent smallpox. The three cases at 50 Walnut street are varioloid. No other suspicious cases reported.

The Board of Health appeals to the people of Atlanta to be thoroughly vaccinated immediately, and thereby present an effectual barrier to any further spread of the disease.

An official bulletin will be issued daily by the Board of Health, giving the exact status of affairs.

JAMES F. ALEXANDER, M. D., President.
JAMES C. AVERY, Secretary.
FLOYD W. MCRAE, M. D.,
for Board of Health.

Vaccination with bovine virus is being carried out by a corps of physicians from house to house, and families are undergoing vaccination by their own physicians to an extent which ought to arrest the progress of the disease. But a most unfortunate attitude has been taken by the *Evening Journal*, calculated to lessen the interest of the people in availing themselves of vaccination. This course is based upon mistaken views of the true interests of Atlanta, and the pages of the paper are closed against a correct statement of the facts.

There was a time when humanized virus might be charged with introducing contamination into the healthy system, but the use of the cowpox or bovine virus is free from any such allegation, and affords either complete protection or such modification, as to secure those vaccinated from any serious consequences of exposure to even the most virulent form of smallpox.

It is a recognized fact that vaccine virus is obtained from animals having a specific disease, and not from a modification resulting from inoculation with variolus matter, as has been erronously supposed by some not properly informed on this subject. If the heifers employed for this purpose are otherwise healthy, the cowpox affords a vaccine matter free from contamination, and there need be no apprehension of contracting any other disease from its introduction into the human body.

In regard to the length of time the subject is protected by vaccination, there is some doubt, and yet it is held to be certainly effective from seven to ten years. It is a wise precaution to repeat vaccination until it ceases to have any effect, and afterwards vaccinate at intervals of ten years or in less time if exposed to smallpox.

Although I had varioloid many years since when attending smallpox cases, it has been thought prudent to repeat the vaccination from time to time, and the vaccine virus, introduced recently, shows some of its specific effects, indicating now that protection has been diminished by lapse of time in my person. It is a well established fact that exceptional cases occur in which variola even is developed again after an attack of the disease in its virulent form, and hence a recurrence may take place when varioloid has made its appearance after vaccination. But the rule still holds good that one attack of variola or varioloid protects the subject against a recurrence, and that vaccination is effective in preventing this disease if repeated every five or even every ten years.

It is therefore of the greatest importance, that all not previously vaccinated should avail themselves of the opportunity afforded at present, to get the protection of vaccination against any possible exposure to smallpox, either now or hereafter. The repetition in those who have not been vaccinated within five years is expedient, as it gives no inconvenience, if the individual is already protected, and if it is accompanied with its specific results, this demonstrates the need of it for complete security. General vaccination is therefore very desirable, to put a stop to this disease. J. McF. G.

---

WE have more than once deprecated the tendency of some of our regular professional brethren of permitting themselves to be advertised in the daily press in the form of an interview on medical matters not touching the public interest. We wish again to go on record as believing that such a course cannot be too strongly condemned. This is especially true in an age when the country in general and Georgia in particular is overrun with quacks and charlatans and the daily papers filled with disgusting and false advertisements of vaunted medical and surgical skill.

The profession of Georgia put itself on record before the last legislature as advocating higher medical education and the expulsion from her borders of quacks and charlatans. This position comports but ill with the physician appearing in the secular press to describe some operation or else being so run to death that he has not time to tell the half he knows or has done.

Such conduct is in direct opposition to both the spirit and letter of our Code, which says in chapter 2, article 1, section 3: "It is derogatory to the dignity of the profession to resort to public advertisements or private cards . . . . . . . or to publish cases or operations in the daily prints, or *suffer such publications to be made*. . . . . . . These are the ordinary practices of empirics and are highly reprehensible in a regular physician."

---

DR. THEODOR BILLROTH, of Vienna, died February 5, 1894, of heart disease. He was one of the most celebrated surgeons of his day and was an author of great renown. He continued his surgical and literary work almost to his end. He published a work on Pathological Histology in 1858; and another on Classification, Diagnosis and Prognosis of Tumors, translated in 1861, through which he became known to the profession in this country. His lectures on Surgical Pathology, published in 1872 by the Appletons, became a text-book in most medical colleges in America. It is justly claimed that his most striking surgical achievement was the performance of the first successful resection of the pylorus for malignant disease. Pean had previously done the operation without success.—*Exchange.*

